# White Light Emission from Thin-Film Samples of ZnO Nanocrystals, Eu^3+^ and Tb^3+^ Ions Embedded in an SiO_2_ Matrix

**DOI:** 10.3390/ma12121997

**Published:** 2019-06-21

**Authors:** Vivek Mangalam, Kantisara Pita

**Affiliations:** Centre for OptoElectronics and Biophotonics, School of Electrical and Electronic Engineering, Nanyang Technological University (NTU), Block S2, 50 Nanyang Avenue, Singapore 639798, Singapore; vivek014@e.ntu.edu.sg

**Keywords:** zinc oxide nanocrystals, europium, terbium, white light, energy transfer, photoluminescence

## Abstract

In this work, a method was developed to determine the concentration of Eu^3+^ and Tb^3+^ ions in a thin-film sample of SiO_2_, co-doped with ZnO-nanocrystals (ZnO-nc), to produce a sample of any desired colour in the International Commission on Illumination (CIE) colour space. Using this method, a white light emitting sample was fabricated. The thin-film sample combines red, green and blue emissions from the Eu^3+^ ions, Tb^3+^ ions and ZnO-nc, respectively, to create white light or light of any desired colour. The emissions at 614 nm and 545 nm from Eu^3+^ and Tb^3+^ ions, respectively, is due to the energy transfer from the excited ZnO-nc to the rare-earth (RE) ions. In this way, only a single excitation wavelength is needed to excite the ZnO-nc, Eu^3+^ and Tb^3+^ ions in the sample to produce emission of a desired colour from the sample. We developed an empirical 4th-degree polynomial equation to determine the concentrations of Eu^3+^ and Tb^3+^ ions to produce light of any desired colour in the CIE colour space. Based on the above empirical equation, the concentration of Eu^3+^ and Tb^3+^ ions for a white light emitting sample was found to be 0.012 and 0.024 molar fractions, respectively. The white light emission from the sample was confirmed by fabricating the sample using the low-cost sol–gel process. The stimulated emission spectra and the experimental emission spectra of the white light sample fit very well. The results presented in this work are important to develop energy efficient solid state lighting devices.

## 1. Introduction

In recent years, white light emitting diodes have been widely studied [1,2,3,4,5] and developed for various solid state lighting applications. White light emitting diodes play an important role in applications, such as in displays, general lighting and automobile headlights. In most of these applications, the white light is produced either through i) a phosphor conversion ion in which a ultra-violet(UV)-blue light emitting diode (LED) is used along with a yellow phosphor or through ii) a combination of red, green and blue (RGB) LEDs [2].

In this work, we present a simple way to produce white light from thin-film samples containing semiconductor nanocrystal and lanthanide rare-earth (RE) ions. The UV-blue emission from zinc oxide nanocrystals (ZnO-nc) is combined with the red and green emissions from the RE ions, such as Europium (3+) and Terbium (3+), respectively, to produce white light. An advantage of using ZnO-nc, Eu^3+^ and Tb^3+^ ions to produce white light is that a thin-film sample of these materials embedded in SiO_2_ can be easily fabricated using the low-cost sol-gel process. In this material system, the ZnO-nc can be excited electrically or using a single excitation wavelength having photon energy higher than the optical gap, which results in the excitation of the ZnO-nc. These excited nanocrystals can then relax through radiative de-excitation, non-radiative de-excitation and by transferring the energy to excite the Eu^3+^ and Tb^3+^ ions. The excited Eu^3+^ and Tb^3+^ ions, in turn, relax through the radiative de-excitation or non-radiative de-excitation process. The radiative de-excitation from the Eu^3+^ ions, Tb^3+^ ions and ZnO-nc results in the red, green and blue emissions, respectively. 

There have been substantial studies demonstrating the energy transfer specifically from ZnO-nc to Eu^3+^ ions [6,7,8,9,10,11,12,13,14,15,16,17,18] and also from ZnO-nc to Tb^3+^ ions [19,20,21,22,23]. Previous works by our group have also reported on the study of the energy transfer mechanism, contribution and transfer efficiency from various ZnO-nc emission centers to Eu^3+^ ions [17,18,24] and Tb^3+^ ions individually [23]. Interestingly, there are very few works on co-doping both Eu^3+^ ions and Tb^3+^ ions together with ZnO-nc in the same SiO_2_ matrix. One such work on co-doping both Eu^3+^ ions and Tb^3+^ ions with ZnO-nc by Luo et al. was on the energy transfer process and the energy transfer mechanism from ZnO-nc to the two RE ions [25]. In the study by Luo et al. the energy transfer between the two RE ions themselves is also investigated, but it does not report on a pure white light emitting sample. In fact, we were unable to find any study where energy transfer from ZnO-nc to Eu^3+^ ions and Tb^3+^ ions was used to create a white light emitting sample.

White light emission from a material system of ZnO-nc and Eu^3+^ ions together with a different RE ion, namely Dy^3+^ (Dysprosium), has been reported by Luo et al. [26]. However, in the work by Luo et al. [26], the ZnO-nc are fabricated in powder form and the RE ion are embedded in the ZnO-nc. The nanocrystals are not embedded in a protective dialectic medium, such as SiO_2_. Furthermore, the white light sample reported in the work by Luo et al. [26] does not have pure white light emission (International Commission on Illumination (CIE) chromaticity coordinate x = y = z = 0.333). In general, we observe that the procedure to systematically obtain the concentration of the different constituent material in the white light emitting sample has not been reported. 

In this work, we fabricate thin-film samples of ZnO-nc in SiO_2_ co-doped with both Eu^3+^ ions and Tb^3+^ ions using the low-cost sol-gel process to create a pure white light emitting sample that can be excited using a single excitation wavelength. The concentration of Eu^3+^ and Tb^3+^ ions in the white light emitting sample is systematically obtained in this work. This is a continuation of our previous works [17,18,23,24]. The white light emission was achieved by first fabricating various thin-film samples of ZnO-nc in SiO_2_ with varying concentrations of Eu^3+^ and Tb^3+^ ions. The photoluminescence (PL) emission intensities from these samples were then used to obtain an empirical equation for the emission intensity as a function of Eu^3+^ ions and Tb^3+^ ions concentration. The empirical emission intensity equation was subsequently used to deduce the concentration of Eu^3+^ ions and Tb^3+^ ions, which give white light emission and orangish-white light emission. Using the deduced values of the Eu^3+^ and Tb^3+^ ion concentration, a white light emitting sample and a orangish-white light emitting sample were then fabricated using the sol-gel process to experimentally confirm the deduction. A white light emission and an orangish-white light emission from the two samples were demonstrated in this work. The knowledge and understanding gained from this work will be beneficial for the development of energy efficient solid state white lighting devices which use the energy transfer process from ZnO-nc to Eu^3+^ and Tb^3+^ ions, to produce light.

## 2. Materials and Method

In this work, the sol-gel process was employed in the fabrication of the thin-film samples due to the low-cost nature of this chemical process. Twenty-six different samples of ZnO nanocrystals (ZnO-nc) embedded in SiO_2_ matrix co-doped with varying concentration of Eu^3+^ ions and Tb^3+^ ions were fabricated, which are listed in Table 1. The samples in this work are labelled as Eu^3+^*_m_*: Tb^3+^*_n_*:ZnO-nc:SiO_2_ where *m* is the molar fraction of the concentration of Eu^3+^ ions calculated using the formula $m=\frac{moles\;of\;\left(Eu^{3+}\right)}{moles\;of\;\left(Eu^{3+}+Tb^{3+}+Zn+Si\right)}$ and *n* is the molar fraction of the concentration of Tb^3+^ ions calculated using the formula $n=\frac{moles\;of\;\left(Tb^{3+}\right)}{moles\;of\;\left(Eu^{3+}+Tb^{3+}+Zn+Si\right)}$. Note here that eight of theses samples are the samples from our previous work which have been reported elsewhere [17,23], while the remaining eighteen samples were newly fabricated for this current work as indicated in Table 1. Furthermore, out of the total twenty-six different samples listed in Table 1, twenty-four of the samples were used to establish the empirical equation in this work, while the remaining two samples (Eu^3+^_0.012_:Tb^3+^_0.024_:ZnO-nc:SiO_2_ and Eu^3+^_0.014_:Tb^3+^_0.084_:ZnO-nc:SiO_2_) were used to confirm the empirical equation.

The samples were fabricated using an identical recipe described in our previous publications [17,18,23,24]. In all the twenty-six samples, the molar ratio of Zn:Si in the samples was maintained at 1:2. The Eu^3+^ ions and Tb^3+^ ions are doped into the SiO_2_ matrix by mixing Europium(III) nitrate pentahydrate and Terbium(III) nitrate pentahydrate salts, respectively, in the SiO_2_ sol. After a 24 h ageing of the two different sols, namely SiO_2_ sol and ZnO-nc sol, they were mixed together before being spin-coated on a Si substrate. These samples were then made into densified thin films by soft baking at 100 °C, followed by annealing at 450 °C using rapid thermal processing (RTP).

The thin-film samples were characterised by studying the photoluminescence (PL) emissions from the sample using SPEX Fluorolog-3 Model FL3-11 spectrofluorometer(Horiba, Edison, NJ, USA). The PL spectra were obtained by exciting the samples at 325 nm using the 450-W xenon short arc lamp of the spectrofluorometer attached to a monochromator and then measuring the emission intensity from the sample at each wavelength ranging from 340 nm to 635 nm using a photomultiplier tube (PMT) detector coupled to another monochromator.

## 3. Results and Discussion

**Figure 1:**
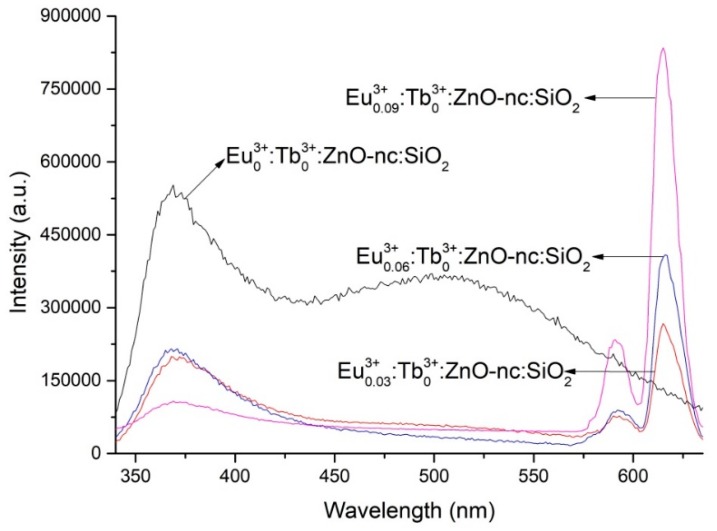
The photoluminescence (PL) spectra of Eu^3+^_0_:Tb^3+^_0_:ZnO-nc:SiO_2_, Eu^3+^_0.03_:Tb^3+^_0_:ZnO-nc:SiO_2_, Eu^3+^_0.06_:Tb^3+^_0_:ZnO-nc:SiO_2_ and Eu^3+^_0.09_:Tb^3+^_0_:ZnO-nc:SiO_2_ samples upon excitation using 325 nm excitation source.

**Figure 2:**
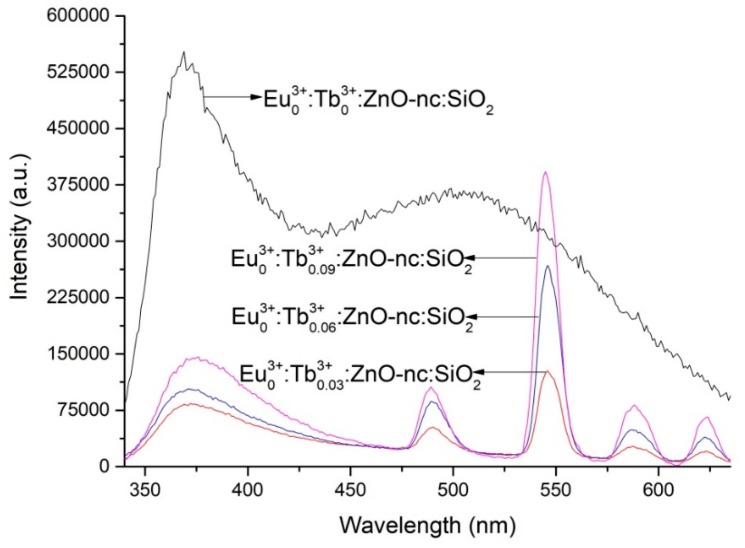
The PL spectra of Eu^3+^_0_:Tb^3+^_0_:ZnO-nc:SiO_2_, Eu^3+^_0_:Tb^3+^_0.03_:ZnO-nc:SiO_2_, Eu^3+^_0_:Tb^3+^_0.06_:ZnO-nc:SiO_2_ and Eu^3+^_0_:Tb^3+^_0.09_:ZnO-nc:SiO_2_ samples upon excitation using 325 nm excitation source.

**Figure 3:**
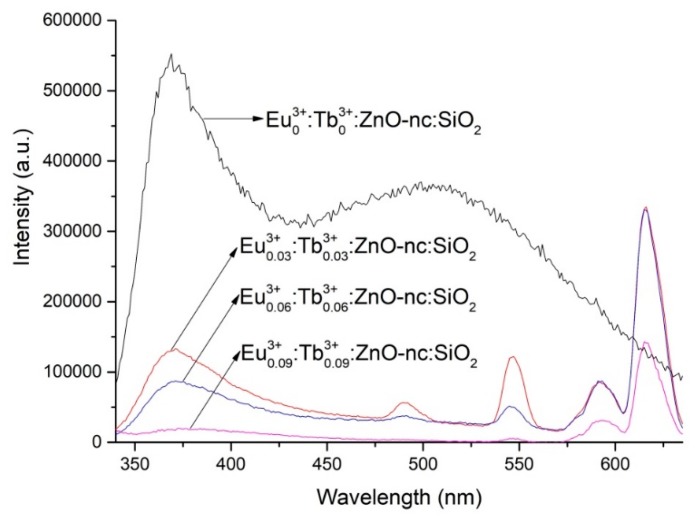
The PL spectra of Eu^3+^_0_:Tb^3+^_0_:ZnO-nc:SiO_2_, Eu^3+^_0.03_:Tb^3+^_0.03_:ZnO-nc:SiO_2_, Eu^3+^_0.06_:Tb^3+^_0.06_:ZnO-nc:SiO_2_ and Eu^3+^_0.09_:Tb^3+^_0.09_:ZnO-nc:SiO_2_ samples upon excitation using 325 nm excitation source.

As mentioned above, to obtain the right concentrations of Eu^3+^ and Tb^3+^ ions together with the ZnO-nc (which is fixed in this work), we need to establish an empirical equation of the PL intensity as a function of Eu^3+^ and Tb^3+^ ion concentrations. The empirical equation is derived based on the PL intensity data of the samples listed in Table 2. We show the PL spectra of some of the samples listed in Table 2 and Figure 1, Figure 2 and Figure 3 below. Figure 1 shows the PL spectra of the SiO_2_ films doped with ZnO-nc and varying concentrations of Eu^3+^ ions, namely Eu^3+^_0.03_:Tb^3+^_0_:ZnO-nc:SiO_2_, Eu^3+^_0.06_:Tb^3+^_0_:ZnO-nc:SiO_2_, Eu^3+^_0.09_:Tb^3+^_0_:ZnO-nc:SiO_2_ and the PL spectra of Eu^3+^_0_:Tb^3+^_0_:ZnO-nc:SiO_2_ sample (the SiO_2_ film doped with ZnO-nc only). These samples of ZnO-nc and Eu^3+^ ions embedded in SiO_2_ are the newly fabricated samples made for this current work (indicated in Table 1). The PL spectra of the other samples doped only with Eu^3+^ ions and ZnO-nc in SiO_2_, namely Eu^3+^_0.04_:Tb^3+^_0_:ZnO-nc:SiO_2_, Eu^3+^_0.08_:Tb^3+^_0_:ZnO-nc:SiO_2_, Eu^3+^_0.12_:Tb^3+^_0_:ZnO-nc:SiO_2_ and Eu^3+^_0.16_:Tb^3+^_0_:ZnO-nc:SiO_2_ samples have been previously published by our group [17] and hence, not shown in Figure 1. From the PL emission spectra, we clearly see the signature emission peaks of Eu^3+^ ions at 590 nm and 614 nm for the samples with Eu^3+^ ions, corresponding to ^5^D_0_→^7^F_1_ and ^5^D_0_→^7^F_2_ transitions, respectively. These emissions which are due to the energy transfer process have been discussed in detail in our previous publication [17]. The PL spectrum of the SiO_2_ film containing only ZnO-nc (Eu^3+^_0_:Tb^3+^_0_:ZnO-nc:SiO_2_ sample), which is also included in Figure 1, shows the characteristic broadband emission of ZnO-nc ranging from 350 nm to 600 nm. The origin of the broadband emission from ZnO-nc together with a detailed discussion on the energy transfer process and contribution of energy transfer from ZnO-nc to Eu^3+^ ion has been reported in our group’s earlier work [17].

Figure 2 shows the PL spectra of the SiO_2_ films doped with ZnO-nc and varying concentrations of Tb^3+^ ions, namely Eu^3+^_0_:Tb^3+^_0.03_:ZnO-nc:SiO_2_, Eu^3+^_0_:Tb^3+^_0.06_:ZnO-nc:SiO_2_ and Eu^3+^_0_:Tb^3+^_0.09_:ZnO-nc:SiO_2_. Again, these samples of ZnO-nc and Tb^3+^ ions embedded in SiO_2_ are the newly fabricated samples made for this current work (indicated in Table 1). The PL spectra of the SiO_2_ film doped only with ZnO-nc (Eu^3+^_0_:Tb^3+^_0_:ZnO-nc:SiO_2_ sample) is also included the figure. The PL spectra of the other samples doped only with Tb^3+^ ions and ZnO-nc in SiO_2_, namely Eu^3+^_0._:Tb^3+^_0.04_:ZnO-nc:SiO_2_, Eu^3+^_0_:Tb^3+^_0.08_:ZnO-nc:SiO_2_, Eu^3+^_0_:Tb^3+^_0.12_:ZnO-nc:SiO_2_ and Eu^3+^_0_:Tb^3+^_0.16_:ZnO-nc:SiO_2_ samples have been published in our earlier work [23] and hence, not shown in Figure 2. Once again from the PL spectra, we observe the characteristic emission from the Tb^3+^ ions at 489 nm, 545 nm, 586 nm and 621 nm in samples with Tb^3+^ ions. These emissions correspond to ^5^D_4_→^7^F_6_, ^5^D_4_→^7^F_5_, ^5^D_4_→^7^F_4_ and ^5^D_4_→^7^F_3_ transitions, respectively. This energy transfer process and mechanism of energy transfer from ZnO-nc to Tb^3+^ ions has been reported in detail in our previous publication [23].

The main focus of this study, as mentioned above, is to demonstrate a white light emitting sample by incorporating both Eu^3+^ ions and Tb^3+^ ions together with ZnO-nc in thin-film samples of SiO_2_. Hence, samples with varying concentrations of both Eu^3+^ ions and Tb^3+^ ions together with a fixed concentration of ZnO-nc in SiO_2_ matrix were also fabricated. Figure 3 shows the PL spectra of a some of the samples of SiO_2_ doped with Eu^3+^ ions, Tb^3+^ ions and ZnO-nc, namely Eu^3+^_0.03_:Tb^3+^_0.03_:ZnO-nc:SiO_2_, Eu^3+^_0.06_:Tb^3+^_0.06_:ZnO-nc:SiO_2_ and Eu^3+^_0.09_:Tb^3+^_0.09_:ZnO-nc:SiO_2_. Again, we also include the PL spectra of the sample doped only with ZnO-nc (Eu^3+^_0_:Tb^3+^_0_:ZnO-nc:SiO_2_ sample). In all of the samples with both Eu^3+^ ions and Tb^3+^ ions co-doped with ZnO-nc in SiO_2_, we clearly observe the emission peaks from Tb^3+^ ions at 489 nm and 545 nm, the emission peaks from Eu^3+^ ions at 590 nm and 614 nm and the relatively broader emission from ZnO-nc with peak intensity at 372 nm. These emissions from the Tb^3+^ and Eu^3+^ ions are the radiative de-excitations of the RE ions excited through the energy transfer process from excited ZnO-nc.

To demonstrate a sample of desired colour (white light in this study), first, the physiologically perceived colour of the samples need to be deduced. This is achieved by calculating the *xyz* chromaticity coordinates of the PL emission from the samples. The *xyz* chromaticity coordinates are a standard defined by the International Commission on Illumination (CIE) which consists of three values denoted by *x*, *y* and *z*. The CIE *x*, *y* and *z* chromaticity coordinates are essentially normalised fractions which give a quantitative relation between the spectrum of emission from the sample and the colour perceived by the three different receptors in the human eye [27]. CIE *x*, *y* and *z* chromaticity values are obtained using the following mathematical equations:

(1) $$X=\overset{\infty }{\underset{0}{\int }}I\left(\lambda \right)\bar{x}\left(\lambda \right)d\lambda$$

(2) $$Y=\overset{\infty }{\underset{0}{\int }}I\left(\lambda \right)\bar{y}\left(\lambda \right)d\lambda$$

(3) $$Z=\overset{\infty }{\underset{0}{\int }}I\left(\lambda \right)\bar{z}\left(\lambda \right)d\lambda$$

(4) $$x=\frac{X}{X+Y+Z}$$

(5) $$y=\frac{Y}{X+Y+Z}$$

(6) $$z=\frac{Z}{X+Y+Z}=1-x-y$$

where I(λ) is the emission intensity of the thin-film sample as a function of wavelength and $\bar{x}\left(\lambda \right),\;\bar{y}\left(\lambda \right)$ and $\bar{z}\left(\lambda \right)$ are the CIE colour-matching functions for the 1964 standard colorimetric observer which are essentially the spectral response of the three different receptors in the human eye [27]. The *x*, *y* and *z* values, correspond to the degree of red, green and blue colours, respectively, as perceived by the human eye. This means, the greater the value of *x*, the more red the sample looks. Similarly, the greater the value of *y*, the more green the sample looks and the greater the value of *z*, the more blue the sample looks.

**Figure 4:**
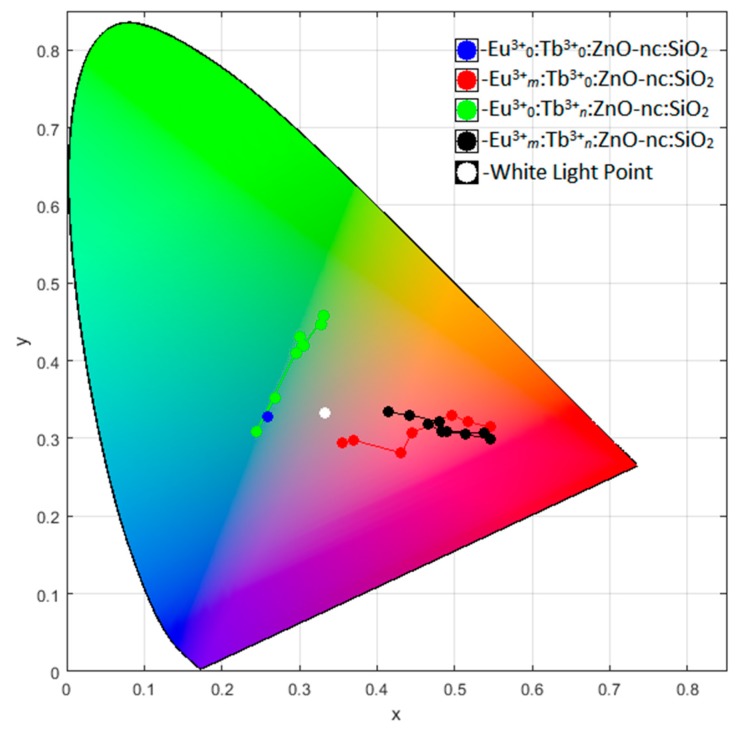
International Commission on Illumination (CIE) 1964 colour space plot with *x* and *y* points of Eu^3+^_0_:Tb^3+^_0_:ZnO-nc:SiO_2_, Eu^3+^*_m_*:Tb^3+^_0_:ZnO-nc:SiO_2_, Eu^3+^_0_:Tb^3+^*_n_*:ZnO-nc:SiO_2_ and Eu^3+^*_m_*:Tb^3+^*_n_*:ZnO-nc:SiO_2_ samples along with the white light point.

Table 2 shows the *x*, *y* and *z* values for the twenty-four samples used to establish the empirical equation in this work, calculated using the above Equations (1)–(6), which is also graphically represented in CIE 1964 colour space plot shown in Figure 4.

After showing, the colour of our samples in the CIE colour space in Figure 4, we will now describe the method to obtain the concentrations of Eu^3+^ and Tb^3+^ ions (ZnO-nc is fixed in this study) to get a white emission which corresponds to x=y=z=0.333. This is the main focus of this paper. To do this, we need to establish an empirical equation to model the emission intensity spectra of the Eu^3+^*_m_*:Tb^3+^*_n_*:ZnO-nc:SiO_2_ samples as a function of Eu^3+^ and Tb^3+^ ion concentrations based on the PL spectra of the samples listed in Table 2. The empirical equation is used here, since the emission of Eu^3+^*_m_*:Tb^3+^*_n_*:ZnO-nc:SiO_2_ samples is a complex process which involves energy transfer from ZnO-nc to the two RE ions and the energy transfer among the RE ions and interactions among all the constituents (RE ions and ZnO-nc). The empirical equation was developed based on the PL spectra of the samples listed in Table 2 which include 1) sample with only ZnO-nc in SiO_2_, 2) samples with Eu^3+^ ions and ZnO-nc in SiO_2_, 3) samples with Tb^3+^ ions and ZnO-nc in SiO_2_ and 4) samples with both Eu^3+^ and Tb^3+^ ions together with ZnO-nc in SiO_2_. Thus, the polynomial equations accounts for the interactions among all the constituents (RE ions and ZnO-nc). 

The intensity spectra of the Eu^3+^*_m_*:Tb^3+^*_n_*:ZnO-nc:SiO_2_ samples can be described by using a polynomial equation in *m* (the molar fraction of the concentration of Eu^3+^ ions) and *n* (the molar fraction of the concentration of Tb^3+^ ions). In this work, the 3rd, 4th and 5th-degree polynomial equation in two variables were considered to model the PL emission of Eu^3+^*_m_*:Tb^3+^*_n_*:ZnO-nc:SiO_2_ samples. The 1st and 2nd-degree polynomial equation in two variables were not considered in this work as the simulated intensity spectra obtained from these equations (not shown) does not show good fit with the experimentally obtained emission intensity values of the sample. We found that the 4th-degree polynomial equation in two variables is optimum to represent the intensity spectra of the Eu^3+^*_m_*:Tb^3+^*_n_*:ZnO-nc:SiO_2_ samples and the justification for this is given below. Mathematically the 4th-degree polynomial equation is written as

(7) $$\begin{array}{ll} I\left(\lambda ,m,n\right)= & C_{00}\left(\lambda \right)+C_{10}\left(\lambda \right)m+C_{01}\left(\lambda \right)n+C_{20}\left(\lambda \right)m^{2}+C_{11}\left(\lambda \right)mn+C_{02}\left(\lambda \right)n^{2}\\ & +C_{30}\left(\lambda \right)m^{3}+C_{21}\left(\lambda \right)m^{2}n+C_{12}\left(\lambda \right)mn^{2}+C_{03}\left(\lambda \right)n^{3}+C_{40}\left(\lambda \right)m^{4}\\ & +C_{31}\left(\lambda \right)m^{3}n+C_{22}\left(\lambda \right)m^{2}n^{2}+C_{13}\left(\lambda \right)mn^{3}+C_{04}\left(\lambda \right)n^{4}, \end{array}$$

where I(λ,m,n) is the emission intensity of the Eu^3+^_m_:Tb^3+^_n_:ZnO-nc:SiO_2_ sample at wavelength λ and $C_{00}\left(\lambda \right),\;C_{10}\left(\lambda \right),C_{01}\left(\lambda \right),C_{20}\left(\lambda \right),C_{11}\left(\lambda \right),C_{02}\left(\lambda \right),C_{30}\left(\lambda \right),C_{21}\left(\lambda \right),C_{12}\left(\lambda \right),$ $C_{03}\left(\lambda \right),C_{40}\left(\lambda \right),C_{31}\left(\lambda \right),C_{22}\left(\lambda \right),C_{13}\left(\lambda \right)$ and $C_{04}\left(\lambda \right)$ are the coefficients of the polynomial equation in *m* and *n*, which are unique for each wavelength λ.

All the values of the coefficient of the polynomial in Equation (7), i.e., the values of all *C*, from $C_{00}\left(\lambda \right)$ to $C_{04}\left(\lambda \right)$, for each emission wavelength (λ) ranging from 340 nm to 635 nm were obtained using the MATLAB parametric fitting optimisation function which employs the linear least squares regression method to find these optimum values. The goodness of the fit of Equation (7) was confirmed by comparing the experimentally obtained PL spectra of the twenty-four samples with those from the empirical Equation (7), which we will refer to as simulated spectra. 

To justify that the 4th-degree polynomial equation in two variables is optimum to represent the intensity spectra of the Eu^3+^*_m_*:Tb^3+^*_n_*:ZnO-nc:SiO_2_ samples, we first calculated the sum of the squares of the difference between the experimental emission intensity value and the simulated emission intensity value at each wavelength, which is known as residual sum of squares and denoted by RSS. The RSS value was calculated for each of 3rd, 4th and 5th-degree polynomial equations. RSS is mathematically is given by

(8) $$RSS=\sum_{\lambda =340}^{635}{\left(I_{E}\left(\lambda \right)-I_{S}\left(\lambda \right)\right)}^{2},$$

where $I_{E}\left(\lambda \right)$ is the experimental emission intensity value and $I_{S}\left(\lambda \right)$ is the simulated emission intensity value. RSS for 3rd, 4th and 5th-degree polynomial equation are denoted by RSS_3_, RSS_4_ and RSS_5_, respectively. To determine which polynomial equations is optimum to simulate the emission spectra, we then calculate and compare the percentage change of RSS from 3rd degree to 4th degree (denoted by %RSS(3→4)) and 4th degree to 5th degree (denoted by %RSS(4→5)). This is given by:

(9) $$\%RSS\left(3\to 4\right)=\frac{RSS_{4}-RSS_{3}}{RSS_{3}}\times 100\%,$$

(10) $$\%RSS\left(4\to 5\right)=\frac{RSS_{5}-RSS_{4}}{RSS_{4}}\times 100\%.$$

The %RSS(3→4) and %RSS(4→5) values are given in Table 3. 

**Figure 5:**
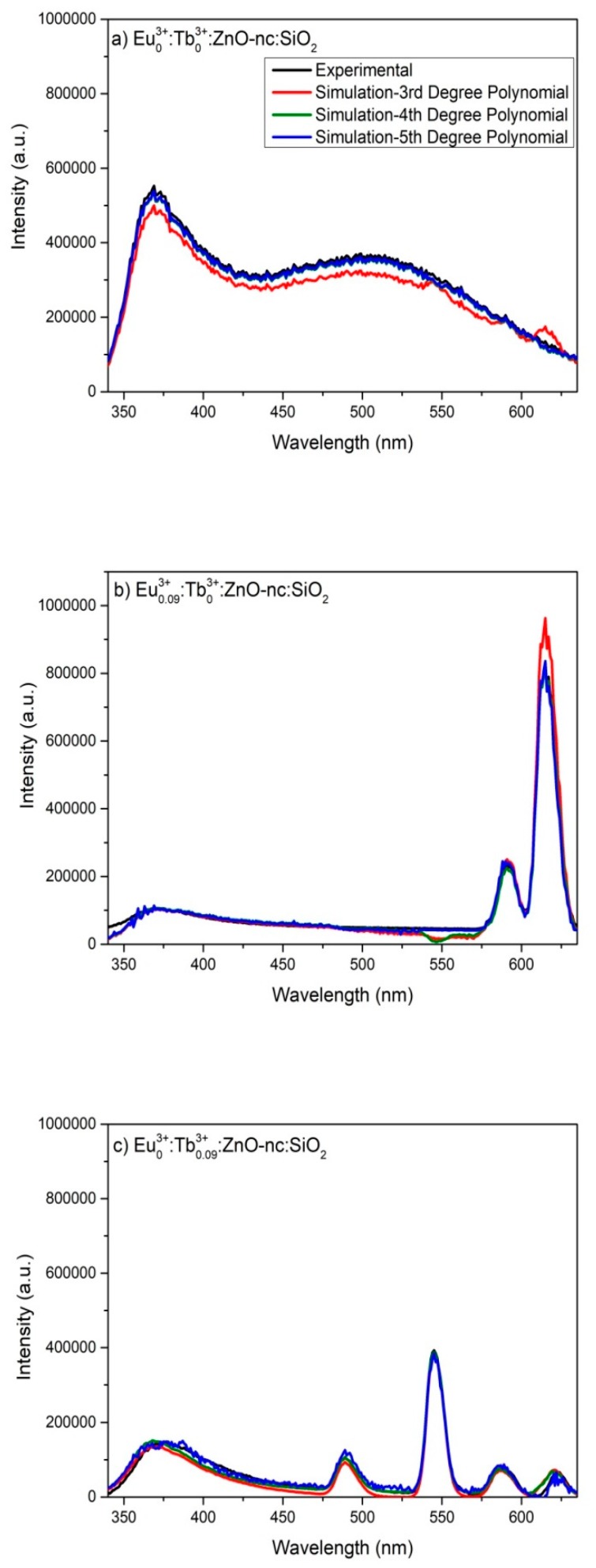

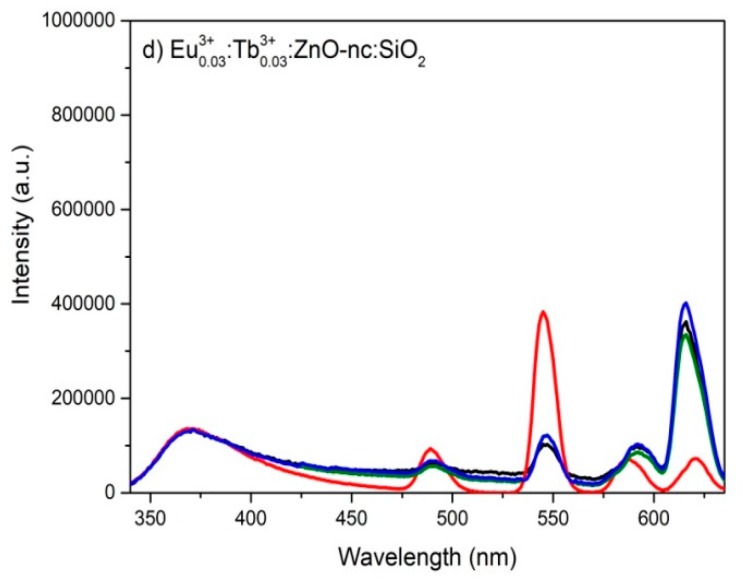
The simulated and experimental emission intensity spectra of four Eu^3+^*_m_*:Tb^3+^*_n_*:ZnO-nc:SiO_2_ samples, namely (**a**) Eu^3+^_0_:Tb^3+^_0_:ZnO-nc:SiO_2_; (**b**) Eu^3+^_0.09_:Tb^3+^_0_:ZnO-nc:SiO_2_; (**c**) Eu^3+^_0_:Tb^3+^_0.09_:ZnO-nc:SiO_2_ and (**d**) Eu^3+^_0.03_:Tb^3+^_0.03_:ZnO-nc:SiO_2_.

Figure 5 shows the simulated intensity spectra obtained using the 3rd, 4th and 5th-degree polynomial equation in two variables together with the experimental emission intensity spectra of four of the Eu^3+^*_m_*:Tb^3+^*_n_*:ZnO-nc:SiO_2_ samples, namely Eu^3+^_0_:Tb^3+^_0_:ZnO-nc:SiO_2_, Eu^3+^_0.09_:Tb^3+^_0_:ZnO-nc:SiO_2_, Eu^3+^_0_:Tb^3+^_0.09_:ZnO-nc:SiO_2_ and Eu^3+^_0.03_:Tb^3+^_0.03_:ZnO-nc:SiO_2_. We observe that the simulated emission spectra obtained using the 3rd, 4th and 5th-degree polynomial equation have a good fit with the experimental emission intensity values. The 3rd-degree polynomial equation simulated spectra of the Eu^3+^_0.03_:Tb^3+^_0.03_:ZnO-nc:SiO_2_ sample is an exception and does not show good fit as seen in Figure 5d. For the particular sample, the emission peak at 545 nm and 614 nm does not fit well with the experimental emission intensity values.

From Table 3 we see that the %RSS(3→4) vary from 48% to 98%, while %RSS(4→5) are all less than 10% for the four samples presented in Figure 5. This shows that the improvement of the simulated spectra from 4th to 5th-degree polynomial is much smaller compared to the improvement of the simulated spectra from 3rd to 4th-degree polynomial. Hence, the 4th-degree polynomial equation in two variables was chosen to represent the experimental intensity spectra of the Eu^3+^*_m_*:Tb^3+^*_n_*:ZnO-nc:SiO_2_ samples in this work.

**Figure 6:**
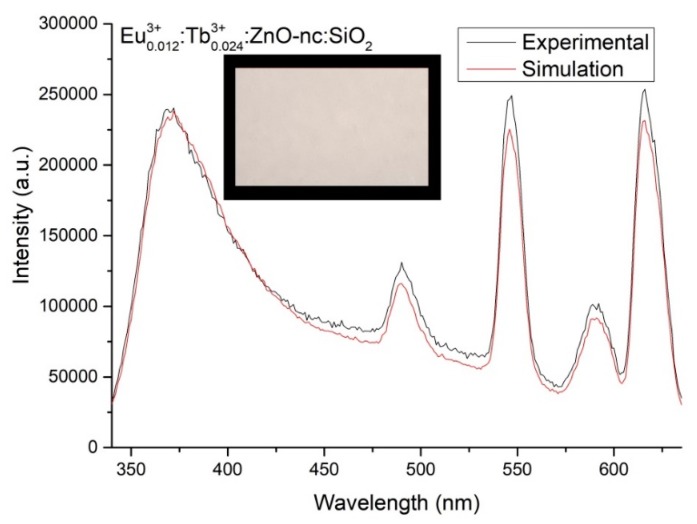
The simulated and experimental emission intensity spectra of Eu^3+^_0.012_:Tb^3+^_0.024_:ZnO-nc:SiO_2_ sample. The inset shows the image of the sample emitting white light upon exciting the sample using 300 nm UV led light.

**Figure 7:**
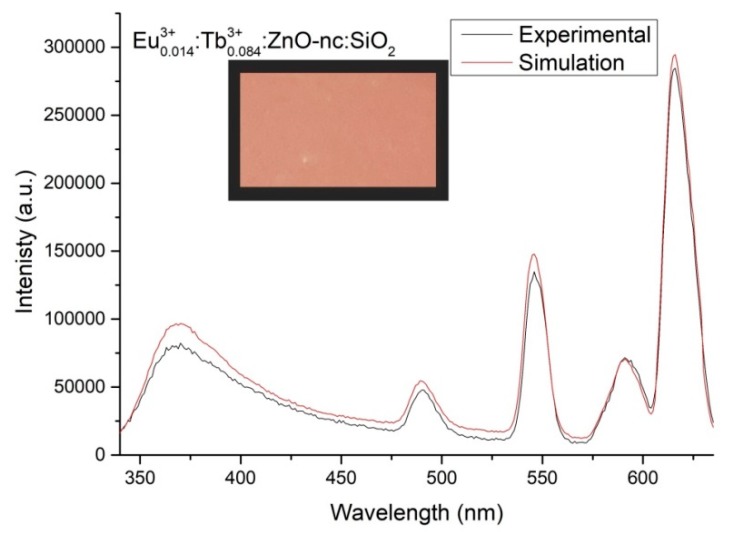
The simulated and experimental emission intensity spectra of Eu^3+^_0.014_:Tb^3+^_0.084_:ZnO-nc:SiO_2_ sample. The inset shows the image of the sample emitting orangish-white light upon exciting the sample using 300 nm UV led light.

Having obtained the empirical equation for the emission intensity of the Eu^3+^*_m_*:Tb^3+^*_n_*:ZnO-nc:SiO_2_ sample as a function of Eu^3+^ ions concentration (m) and Tb^3+^ ions concentration (n), we then used the empirical Equation (7) to generate the emission intensity spectra of Eu^3+^*_m_*:Tb^3+^*_n_*:ZnO-nc:SiO_2_ samples for every value of m and n ranging from 0 to 0.090 with a difference of 0.001. The CIE *x*, *y* and *z* chromaticity values were then calculated for each of the simulated emission intensity using Equations (1) to (6). We found that for a white light emission corresponding to x=y=z=0.333 in the CIE colour space, the values were *m* = 0.012 and *n* = 0.024. We also determined the Eu^3+^ ions and Tb^3+^ ions concentrations for CIE chromaticity values (chosen arbitrarily), x=0.451, y=0.354 and z=0.194, which was found to be m=0.014 and n=0.084. We then fabricated two sample, namely Eu^3+^_0.012_:Tb^3+^_0.024_:ZnO-nc:SiO_2_ for a white light sample and Eu^3+^_0.014_:Tb^3+^_0.084_:ZnO-nc:SiO_2_ sample. The measured PL spectra are shown in Figure 6 and Figure 7, respectively. As expected, we obtained a white light emission from the white light sample, Eu^3+^_0.012_:Tb^3+^_0.024_:ZnO-nc:SiO_2_, as shown in Figure 6. Figure 6 and Figure 7 also shows that the stimulated and the experimental spectra fit very well. In short, we have successfully demonstrated that we are able to design and fabricate Eu^3+^*_m_*:Tb^3+^*_n_*:ZnO-nc:SiO_2_ samples which emit light of any desired colours in the CIE colour space, using a single wavelength excitation. 

## 4. Conclusions

In conclusion, in this work we have developed a method to determine the concentration of Eu^3+^ ions and Tb^3+^ ions in a thin-film sample of SiO_2_ co-doped with ZnO-nc (Eu^3+^*_m_*:Tb^3+^*_n_*:ZnO-nc:SiO_2_ samples) to produce a white light emitting sample or a sample emitting any desired colour in the CIE colour space. Based on the PL data of our samples having various Eu^3+^ and Tb^3+^ ion concentrations and fixed ZnO-nc concentration, we established a 4th-degree polynomial equation in *m* (the molar fraction of the concentration of Eu^3+^ ions) and *n* (the molar fraction of the concentration of Tb^3+^ ions) to determine the values of *m* and *n* to produce a white light or any desired colour emitting sample. The sample combines the red, green and blue emissions from the Eu^3+^ ions, Tb^3+^ ions and ZnO-nc, respectively, to create the white light or any desired colour emission. The emissions from Eu^3+^ ions and Tb^3+^ ions are due to the energy transfer from ZnO-nc to the RE ions. Hence, only a single excitation wavelength, which is used to excite the ZnO-nc, is required to produce various colour emissions. For a white light emitting sample, the values of *m* and *n* are found to be 0.012 and 0.024, respectively. Similarly, for a sample with CIE chromaticity values (chosen arbitrarily), x=0.451, y=0.354 and z=0.194, the values of *m* and *n* correspond to 0.014 and 0.084, respectively. We showed that the stimulated and the experimental spectra of these two samples fit very well. The Eu^3+^_0.012_:Tb^3+^_0.024_:ZnO-nc:SiO_2_ sample, indeed, gives a white light emission. The results presented in this work are important to develop energy efficient solid state lighting devices of any desired colour, such as white light emission, using a single excitation wavelength. In general, this knowledge will help in the development of devices using semiconductor nanocrystals as sensitisers to excite RE ions which have wide ranging application in lasers, optical amplifiers, lighting and display technologies.

## Figures and Tables

**Table 1:** List of 26 samples fabricated using the sol-gel process.

Samples from Previous Work	Samples Newly Fabricated for Current Work
Eu^3+^_0.04_:Tb^3+^_0_:ZnO-nc:SiO_2_	Eu^3+^_0_:Tb^3+^_0_:ZnO-nc:SiO_2_
Eu^3+^_0.08_:Tb^3+^_0_:ZnO-nc:SiO_2_	Eu^3+^_0.03_:Tb^3+^_0_:ZnO-nc:SiO_2_
Eu^3+^_0.12_:Tb^3+^_0_:ZnO-nc:SiO_2_	Eu^3+^_0.06_:Tb^3+^_0_:ZnO-nc:SiO_2_
Eu^3+^_0.16_:Tb^3+^_0_:ZnO-nc:SiO_2_	Eu^3+^_0.09_:Tb^3+^_0_:ZnO-nc:SiO_2_
Eu^3+^_0_:Tb^3+^_0.04_:ZnO-nc:SiO_2_	Eu^3+^_0_:Tb^3+^_0.03_:ZnO-nc:SiO_2_
Eu^3+^_0_:Tb^3+^_0.08_:ZnO-nc:SiO_2_	Eu^3+^_0_:Tb^3+^_0.06_:ZnO-nc:SiO_2_
Eu^3+^_0_:Tb^3+^_0.12_:ZnO-nc:SiO_2_	Eu^3+^_0_:Tb^3+^_0.09_:ZnO-nc:SiO_2_
Eu^3+^_0_:Tb^3+^_0.16_:ZnO-nc:SiO_2_	Eu^3+^_0.03_:Tb^3+^_0.03_:ZnO-nc:SiO_2_
-	Eu^3+^_0.03_:Tb^3+^_0.06_:ZnO-nc:SiO_2_
-	Eu^3+^_0.03_:Tb^3+^_0.09_:ZnO-nc:SiO_2_
-	Eu^3+^_0.06_:Tb^3+^_0.03_:ZnO-nc:SiO_2_
-	Eu^3+^_0.06_:Tb^3+^_0.06_:ZnO-nc:SiO_2_
-	Eu^3+^_0.06_:Tb^3+^_0.09_:ZnO-nc:SiO_2_
-	Eu^3+^_0.09_:Tb^3+^_0.03_:ZnO-nc:SiO_2_
-	Eu^3+^_0.09_:Tb^3+^_0.06_:ZnO-nc:SiO_2_
-	Eu^3+^_0.09_:Tb^3+^_0.09_:ZnO-nc:SiO_2_
-	Eu^3+^_0.012_:Tb^3+^_0.024_:ZnO-nc:SiO_2_
-	Eu^3+^_0.014_:Tb^3+^_0.084_:ZnO-nc:SiO_2_

**Table 2:** International commission on illumination (CIE) *x*, *y* and *z* chromaticity values of the twenty-four different samples used to establish the empirical equation of the PL intensity as a function of Eu^3+^ and Tb^3+^ ion concentrations.

Samples	*X*	*Y*	*Z*
Eu^3+^_0_:Tb^3+^_0_:ZnO-nc:SiO_2_	0.258	0.328	0.414
Eu^3+^_0.03_:Tb^3+^_0_:ZnO-nc:SiO_2_	0.356	0.294	0.35
Eu^3+^_0.04_:Tb^3+^_0_:ZnO-nc:SiO_2_	0.369	0.298	0.333
Eu^3+^_0.06_:Tb^3+^_0_:ZnO-nc:SiO_2_	0.4313	0.281	0.288
Eu^3+^_0.08_:Tb^3+^_0_:ZnO-nc:SiO_2_	0.444	0.306	0.25
Eu^3+^_0.09_:Tb^3+^_0_:ZnO-nc:SiO_2_	0.496	0.33	0.174
Eu^3+^_0.12_:Tb^3+^_0_:ZnO-nc:SiO_2_	0.517	0.321	0.162
Eu^3+^_0.16_:Tb^3+^_0_:ZnO-nc:SiO_2_	0.546	0.314	0.14
Eu^3+^_0_:Tb^3+^_0.03_:ZnO-nc:SiO_2_	0.268	0.352	0.38
Eu^3+^_0_:Tb^3+^_0.04_:ZnO-nc:SiO_2_	0.244	0.309	0.447
Eu^3+^_0_:Tb^3+^_0.06_:ZnO-nc:SiO_2_	0.295	0.41	0.295
Eu^3+^_0_:Tb^3+^_0.08_:ZnO-nc:SiO_2_	0.301	0.431	0.268
Eu^3+^_0_:Tb^3+^_0.09_:ZnO-nc:SiO_2_	0.305	0.419	0.276
Eu^3+^_0_:Tb^3+^_0.12_:ZnO-nc:SiO_2_	0.332	0.458	0.21
Eu^3+^_0_:Tb^3+^_0.16_:ZnO-nc:SiO_2_	0.328	0.447	0.225
Eu^3+^_0.03_:Tb^3+^_0.03_:ZnO-nc:SiO_2_	0.414	0.334	0.252
Eu^3+^_0.03_:Tb^3+^_0.06_:ZnO-nc:SiO_2_	0.441	0.33	0.229
Eu^3+^_0.03_:Tb^3+^_0.09_:ZnO-nc:SiO_2_	0.481	0.321	0.198
Eu^3+^_0.06_:Tb^3+^_0.03_:ZnO-nc:SiO_2_	0.465	0.318	0.217
Eu^3+^_0.06_:Tb^3+^_0.06_:ZnO-nc:SiO_2_	0.484	0.308	0.208
Eu^3+^_0.06_:Tb^3+^_0.09_:ZnO-nc:SiO_2_	0.538	0.307	0.155
Eu^3+^_0.09_:Tb^3+^_0.03_:ZnO-nc:SiO_2_	0.49	0.309	0.201
Eu^3+^_0.09_:Tb^3+^_0.06_:ZnO-nc:SiO_2_	0.514	0.305	0.181
Eu^3+^_0.09_:Tb^3+^_0.09_:ZnO-nc:SiO_2_	0.545	0.3	0.155

**Table 3:** Percentage change in the RSS value from 3rd-degree to 4th-degree and 4th-degree to 5th-degree polynomial equations.

Samples	Percentage Change	
	RSS%(3→4)	RSS%(4→5)
Eu^3+^_0_:Tb^3+^_0_:ZnO-nc:SiO_2_	90.44%	5.22%
Eu^3+^_0.09_:Tb^3+^_0_:ZnO-nc:SiO_2_	76.27%	9.85%
Eu^3+^_0_:Tb^3+^_0.09_:ZnO-nc:SiO_2_	48.91%	2.64%
Eu^3+^_0.03_:Tb^3+^_0.03_:ZnO-nc:SiO_2_	98.21%	4.48%

## References

[1] Mukherjee S., Thilagar P. (2014). Organic white-light emitting materials. Dyes Pigments.

[2] Chen D., Xiang W., Liang X., Zhong J., Yu H., Ding M., Lu H., Ji Z. (2015). Advances in transparent glass-ceramic phosphors for white light-emitting diodes-a review. J. Eur. Ceram. Soc..

[3] Chen P., Li Q., Grindy S., Holten-Andersen N. (2015). White-light-emitting lanthanide metallogels with tunable luminescence and reversible stimuli-responsive properties. J. Am. Chem. Soc..

[4] Li X., Wu Y., Zhang S., Cai B., Gu Y., Song J., Zeng H. (2016). CsPbX3 quantum dots for lighting and displays: Room-temperature synthesis, photoluminescence superiorities, underlying origins and white light-emitting diodes. Adv. Funct. Mater..

[5] Liu J., Sun W., Liu Z. (2016). White-light emitting materials with tunable luminescence based on steady Eu(III) doping of Tb(III) metal-organic frameworks. RSC Adv..

[6] Ishizumi A., Fujita S., Yanagi H. (2011). Influence of atmosphere on photoluminescence properties of Eu-doped ZnO nanocrystals. Opt. Mater..

[7] Luo L., Huang F.Y., Guo G.J., Tanner P.A., Chen J., Tao Y.T., Zhou J., Yuan L.Y., Chen S.Y., Chueh Y.L. (2012). Efficient doping and energy transfer from ZnO to Eu^3+^ ions in Eu^3+^-doped ZnO nanocrystals. J. Nanosci. Nanotechnol..

[8] Zhang Y., Liu Y., Li X., Wang Q.J., Xie E. (2011). Room temperature enhanced red emission from novel Eu^3+^ doped ZnO nanocrystals uniformly dispersed in nanofibers. Nanotechnology.

[9] Lin T., Zhang X.-W., Wang Y.-J., Xu J., Wan N., Liu J.-F., Xu L., Chen K.-J. (2012). Luminescence enhancement due to energy transfer in ZnO nanoparticles and Eu^3+^ ions co-doped silica. Thin Solid Films.

[10] Kumar V., Kumar V., Som S., Duvenhage M.M., Ntwaeaborwa O.M., Swart H.C. (2014). Effect of Eu doping on the photoluminescence properties of ZnO nanophosphors for red emission applications. Appl. Surf. Sci..

[11] Najafi M., Haratizadeh H. (2015). The effect of growth conditions and morphology on photoluminescence properties of Eu-doped ZnO nanostructures. Solid State Sci..

[12] Najafi M., Haratizadeh H. (2015). Investigation of intrinsic and extrinsic defects effective role on producing intense red emission in ZnO:Eu nanostructures. Mater. Res. Bull..

[13] Pessoni H.V.S., Maia L.J.Q., Franco A. (2015). Eu-doped ZnO nanoparticles prepared by the combustion reaction method: Structural, photoluminescence and dielectric characterization. Mat. Sci. Semicon. Proc..

[14] Singh L. (2015). Photoluminescence studies of ZnO, ZnO:Eu and ZnO:Eu nanoparticles covered with Y_2_O_3_ matrix. Mater. Sci. Appl..

[15] Ntwaeaborwa O.M., Mofokeng S.J., Kumar V., Kroon R.E. (2017). Structural, optical and photoluminescence properties of Eu^3+^ doped ZnO nanoparticles. Spectrochim. Acta A.

[16] Huang J., Liu S., Gao B., Jiang T., Zhao Y., Liu S., Kuang L., Xu X. (2014). Synthesis and optical properties of Eu^3+^ doped ZnO nanoparticles used for white light emitting diodes. J. Nanosci. Nanotechnol..

[17] Mangalam V., Pita K., Couteau C. (2016). Study of energy transfer mechanism from ZnO nanocrystals to Eu^3+^ ions. Nanoscale Res. Lett..

[18] Mangalam V., Pita K. (2017). Energy transfer efficiency from ZnO-nanocrystals to Eu^3+^ ions embedded in SiO_2_ film for emission at 614 nm. Materials.

[19] Kumar P., Yadav A.K., Joshi A.G., Bhattacharyya D., Jha S.N., Pandey P.C. (2018). Influence of Li co-doping on structural property of sol-gel derived terbium doped zinc oxide nanoparticles. Mater. Charact..

[20] Kabongo G.L., Mhlongo G.H., Malwela T., Mothudi B.M., Hillie K.T., Dhlamini M.S. (2014). Microstructural and photoluminescence properties of sol-gel derived Tb^3+^ doped ZnO nanocrystals. J. Alloy. Compd..

[21] Jin N., Li H., Liu F., Xie Y.-H. (2016). Microstructure and luminescence properties of Tb^3+^ doped ZnO quantum dots. J. Nanosci. Nanotechnol..

[22] Kumar V., Som S., Kumar V., Kumar V., Ntwaeaborwa O.M., Coetsee E., Swart H.C. (2014). Tunable and white emission from ZnO:Tb^3+^ nanophosphors for solid state lighting applications. Chem. Eng. J..

[23] Mangalam V., Pita K. Energy transfer from zno nanocrystals to terbium (3+) ions: A spectral overlap study. Proceedings of the 2016 IEEE Photonics Conference (IPC).

[24] Mangalam V., Pita K. (2018). Effect of the interaction distance on 614 nm red emission from Eu^3+^ ions due to the energy transfer from ZnO-nc to Eu^3+^ ions. Opt. Mater. Express.

[25] Luo L., Huang F.Y., Dong G.S., Fan H.H., Li K.F., Cheah K.W., Chen J. (2014). Strong luminescence and efficient energy transfer in Eu^3+^/Tb^3+^-codoped ZnO nanocrystals. Opt. Mater..

[26] Luo L., Huang F.Y., Dong G.S., Wang Y.H., Hu Z.F., Chen J. (2016). White light emission and luminescence dynamics in Eu^3+^/Dy^3+^ codoped zno nanocrystals. J. Nanosci. Nanotechnol..

[27] Hunt R.W.G., Pointer M.R. (2011). Measuring Colour.

